# Overexpression of ANAC046 Promotes Suberin Biosynthesis in Roots of *Arabidopsis thaliana*

**DOI:** 10.3390/ijms20246117

**Published:** 2019-12-04

**Authors:** Kashif Mahmood, Viktoria Valeska Zeisler-Diehl, Lukas Schreiber, Yong-Mei Bi, Steven J. Rothstein, Kosala Ranathunge

**Affiliations:** 1 Department of Molecular and Cellular Biology, University of Guelph, Guelph, ON N1G2W1, Canada; kmahmood@noble.org (K.M.); ybi@uoguelph.ca (Y.-M.B.); rothstei@uoguelph.ca (S.J.R.); 2 Noble Research Institute, Limited Liability Company (LLC), 2510 Sam Noble Parkway, Ardmore, OK 73401, USA; 3 Department of Plant Ecophysiology, Institute of Cellular and Molecular Botany, University of Bonn, Kirschallee 1, 53115 Bonn, Germany; vzeisler@uni-bonn.de (V.V.Z.-D.); lukas.schreiber@uni-bonn.de (L.S.); 4 School of Biological Sciences, University of Western Australia, 35 Stirling Highway, Crawly, Perth, WA 6009, Australia

**Keywords:** *ANAC046*, *Arabidopsis thaliana*, permeability, suberin biosynthesis, transcription activator, roots

## Abstract

NAC (NAM (no apical meristem), ATAF1/2, and CUC2 (cup-shaped cotyledon)) proteins are one of the largest families of plant-specific transcription factors, and this family is present in a wide range of land plants. Here, we have investigated the role of *ANAC046* in the regulation of suberin biosynthesis and deposition in *Arabidopsis*. Subcellular localization and transcriptional activity assays showed that *ANAC046* localizes in the nucleus, where it functions as a transcription activator. Analysis of the P_ANAC046_:GUS lines revealed that *ANAC046* is mainly expressed in the root endodermis and periderm, and is also induced in leaves by wounding. The transgenic lines overexpressing *ANAC046* exhibited defective surfaces on the aerial plant parts compared to the wild-type (WT) as characterized by increased permeability for Toluidine blue stain and greater chlorophyll leaching. Quantitative RT-PCR analysis showed that the expression of suberin biosynthesis genes was significantly higher in the roots and leaves of overexpression lines compared to the WT. The biochemical analysis of leaf cuticular waxes showed that the overexpression lines accumulated 30% more waxes than the WT. Concurrently, overexpression lines also deposited almost twice the amount of suberin content in their roots compared with the WT. Taken together, these results showed that *ANAC046* is an important transcription factor that promotes suberin biosynthesis in *Arabidopsis thaliana* roots.

## 1. Introduction

Given their sessile growth, plants employ protective mechanisms to cope with environmental challenges in their surroundings. Amongst these, the development of lipophilic apoplastic barriers, which are made of cutin and suberin, holds great significance [1]. These barriers provide protection against threats that plants face above- or below-ground during their life [2]. Despite having striking similarities in their biochemical properties, both of these barriers are structurally and functionally distinct as their production and deposition is regulated in a highly spatio-temporal-dependent manners [3,4]. Cutin is the integral part of the plant cuticle and is constitutively produced by the epidermal cells of nearly all the aerially developing plants tissues and thus functions as an interface between plants internal tissues or cell layers and the external environment [2]. In addition to providing structural support, the cuticle protects plants from desiccation and pathogen invasion [2,5,6,7]. Suberin deposition, on the other hand, mainly occurs in the root endodermis and exodermis, leaf bundle sheath, seed coats and chalazal micropyle opening/hilum of seed and tuber under normal growth conditions [2,8]. In roots, suberin deposition is further enhanced by abiotic and biotic stresses such as high salinity, waterlogging and high nutrients [3,9,10]. Moreover, suberin biopolymer also plays an important role in wound healing where induced suberin is deposited around the wounding site to seal the damaged plant tissues [11,12]. Regardless of the sites and factors of its deposition, the primary function of suberin polymer is to regulate the movement of water and solutes across the cell/tissue layers and also to restrict pathogen entry into tissues, thereby ensuring tissue integrity and plant survival.

Despite sharing lots of similarities at the biochemical levels, suberin polymers are unique and distinguishable from cutin polymers based on presence of longer chain fatty acids and aromatic compounds [2,13]. Over the last few years, the characterization of a number of mutants has facilitated the identification of genes, which are involved in the biosynthesis and deposition of suberin polymers in spatio-temporal manners in *Arabidopsis thaliana*. These include proteins/enzymes such as β-ketoacyl-CoA synthase (KCS) to produce very long-chain fatty acids (VLCFA) [14,15]; fatty acyl reductases (FAR1, FAR4, FAR5) to produce alcohols and α,ω-diols [16]; cytochrome P450 enzymes (CYP86A1, CYP86B1) to synthesize ω-hydroxy fatty acids (ω-OHs) and α,ω-dicarboxylic acids (DCAs) [17,18,19]; glycerol 3-phosphate acyltransferases (GPAT5, GPAT7) to synthesize sn-2 monoacylglycerols from acyl transfer to glycerol 3-phosphate [20,21] and BAHD-type acetyltransferases, such as aliphatic suberin feruloyl transferase and fatty alcohol:caffeoyl-coa caffeoyl transferase (ASFT and FACT) involved in linking of aromatic compounds to the aliphatic part of the suberin biopolymer [22,23]. Further details on suberin genomics can be found in the review of Ranathunge et al. (2011). More recently, three members from a subfamily of ATP-Binding Cassette G transporters (ABCG2, ABCG6 and ABCG20) [24], and a member of glycosylphosphatidylinositol (GPI)-anchored lipid transfer proteins (LTPG15) [25], have been identified as being involved in the transport of suberin monomers to the apoplast for suberin lamellae deposition.

A highly spatio-temporal deposition of suberin polymers requires a stringent transcriptional regulation of the genes involved in the biosynthesis, transport and polymerization of suberin monomers. Recently, few genes belonging to the MYB (Myeloblastosis) family of transcription factors (TF) have been identified, which positively regulate suberin biosynthesis in different plant species. For example, overexpression of *AtMYB41* resulted in the ectopic suberization of leaf tissues in *Arabidopsis thaliana*. Given that *AtMYB41* expression and suberin depositions were induced by ABA and drought, a role of stress-induced suberin biosynthesis was proposed for *AtMYB41* [26]. Two paralogous MYB genes, *MYB107* and *MYB9*, have been found to positively regulate suberin biosynthesis in *Arabidopsis* seed coats by directly binding and activating the expression of suberin biosynthesis genes [27,28]. Another member of MYB family in cork oak tree, *QsMYB1*, positively regulates the expression genes involved in suberin biosynthesis where it is directly involved in cork development [29]. However, a member of NAC transcription factors family, *StNAC103*, negatively regulates the accumulation of suberin polyester and associated wax in tuber skin [30].

The NAC TFs belong to an important family of plant-specific TFs which have been shown to regulate diverse biological functions in different plant species such as organ development, lignin biosynthesis, leaf senescence and responses to environmental stimuli [31,32,33,34,35,36,37,38]. In particular, *ANAC046* has, recently, been found to promote senescence in *Arabidopsis* leaves by directly inducing the expression of chlorophyll degradation and senescence associated genes [39], and programmed cell death in the *Arabidopsis* columella and lateral root cap by activating the expression of programmed cell death genes such as *BFN1* and *EXI1* [40]. Using ATTED-II database (http://atted.jp), we found a number of suberin biosynthesis genes that co-expressed with *BFN1* and *EXI1* such as *CYP86B1*, *FAR4*, *FAR5* and *GPAT5*. This led us to hypothesize that ANAC046 may also have a role in the modulation of suberin biosynthesis. Therefore, we aimed to investigate the role of *ANAC046* in the regulation of suberin biosynthesis using transgenic approach in *Arabidopsis thaliana*. Analysis of the P_ANAC046_:GUS lines showed that the *ANAC046* promoter is predominantly active in the endodermis and periderm of roots (a primary site of suberin biosynthesis and deposition), floral buds, young developing and senescing siliques. Moreover, *ANAC046* expression was also greater in senescing leaves and induced upon mechanical wounding in green rosette leaves. Overexpression of *ANAC046* leads to an increased expression of suberin biosynthesis genes in the leaves, which resulted in elevated fatty acids, specifically very-long chain-fatty acids (VLCFA) of C24 and C26 as well as sterols. It also resulted in doubling of the suberin contents in the roots compared to WT. These results suggested that *ANAC046* primarily mediates the biosynthesis of suberin in roots of *Arabidopsis*.

## 2. Results

### 2.1. ANAC046 Localizes in the Nucleus and Acts as a Transcriptional Activator

**Figure 1:**
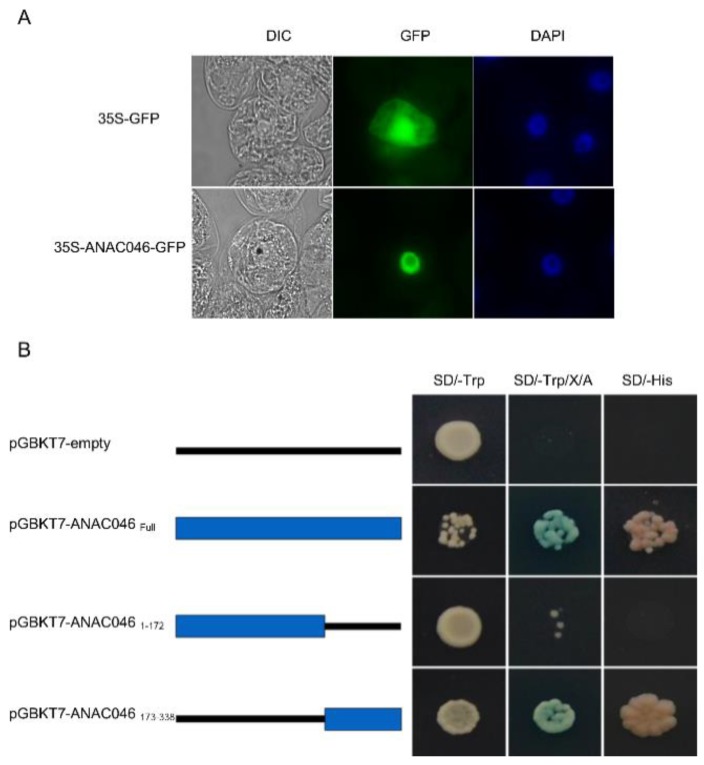
Subcellular localization and transcriptional activity assays. (**A**) Subcellular localization of *ANAC046* determined by transforming Bright Yellow-2 (BY-2) cells with 35:GFP and 35:ANAC046-GFP constructs. The cells were imaged under an epifluorescence microscope. (GFP, green fluorescent protein; DIC, differential interference contrast, DAPI, 4′,6-diamidino-2-phenylindole). (**B**) Transcription activation assay for ANAC046. Sequences encoding full-length protein (1–338 amino acids), N-terminal domain encompassing NAM domain (1–172 amino acids) and C-terminal domain (173–338 amino acids) were cloned individually downstream of the DNA-binding domain (DB) in pGBKT7-DB vector and transformed into Y2Hgold strain. Transcription activity of the constructs was determined on the basis of cloned proteins to activate the expression of reporter genes, and, thereby, allowing the growth of yeast cells on SD/-Trp/X/A and SD/-His plates. SD/-Trp (synthetic defined medium without tryptophan); X (X-α-Gal); A (Aureobasidin A); SD/-His (synthetic defined medium without histidine).

Subcellular localization of *ANAC046* was analyzed by expressing 35S:*ANAC046*-GFP and 35S:GFP constructs in tobacco BY2 (Bright Yellow-2) cells. The BY2 cells transformed with 35:ANAC046-GFP showed GFP (green fluorescent protein) signals only in the nucleus (Figure 1A). In contrast, the BY2 cells transformed with 35S:GFP construct showed GFP signal in both cytosol and nucleus, suggesting that ANAC046 protein has a nuclear localization.

The transcriptional activity of ANAC046 was analyzed by using GAL4 reporter system in yeast. The full-length coding sequence (1–338 amino acids) of *ANAC046* was fused downstream of the Gal4 DNA-binding domain in the pGBKT7-DB vector and expressed in yeast the (Y2HGold strain). Results showed that yeast cells transformed with pGBKT7-*ANAC046* were able to grow on SD/-Trp, SD/-Trp/X/A as well as on SD/-His, suggesting that *ANAC046* activated the expression of reporter genes (Figure 1B). On the contrary, yeast cells transformed with the empty vector could only grow on SD/-Trp plates (Figure 1B). We also determined the activation domain of *ANAC046* protein by expressing the tagged N-terminal (NAM domain from 1–172 amino acids) and C-terminal (from 173–338 amino acids) domains with GAL4 DNA-binding domain (DB) in yeast cells. Only the yeast cells transformed with the C-terminal domain construct (pGBKT7-DB-*ANAC046*_173–338_) could grow on SD/-Trp/X/A and SD/His plates, suggesting that *ANAC046* transcriptional activity lies in its C-terminal domain (Figure 1B).

### 2.2. Spatio-Temporal Expression of ANAC046 in Arabidopsis

**Figure 2:**
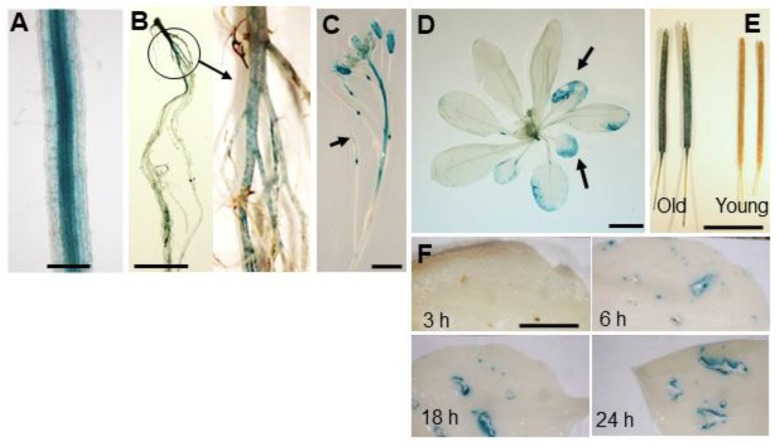
*ANAC046* promoter β-glucuronidase (GUS) activity assay. GUS activity in the (**A**) roots of 12-day-old seedlings grown on ½ MS-agar (1% *w*/*v*) containing 1% sucrose (Bar = 200 µM), (**B**) roots, (**C**) young inflorescence with floral buds and young siliques, (**D**) whole rosette, from 32-day-old soil-grown plants (Bar = 1 cm), (**E**) young/green vs. senescing siliques from 42-day-old soil-grown plants (Bar = 1 cm) and (**F**) young rosette leaves from 21-day-old soil-grown plants after 3, 6, 18 and 24 h of mechanical wounding, of P_ANAC046_::GUS transgenic line (Bar = 0.4 cm).

Spatio-temporal expression of *Arabidopsis* transgenic lines expressing P_ANAC046_:GUS cassette showed that *ANAC046* is mainly expressed in the root endodermis (Figure 2A) and periderm (Figure 2B), floral organs and at the base of young developing siliques (Figure 2C). In the rosette leaves, the β-Glucuronidase (GUS) activity was mainly detected in the older leaves compared to young ones (Figure 2D). Quantitative RT-PCR analysis of green vs senescing leave further confirmed that *ANAC046* could be induced by senescence (Supplementary Figure S1A). A similar age-dependent GUS activity was also detected in the partially senescing/older siliques of 45-day-old plants compared to green/younger plants (Figure 2E). Furthermore, the GUS activity was also induced in the young green leaves of P_ANAC046_:GUS lines by mechanical damage (Figure 2F). At 3 h time-point, no GUS expression appeared in wounded leaves but appeared 6 h after wounding and increased the color intensity further with time, suggesting a wound-induced expression of *ANAC046* in *Arabidopsis* (Figure 2F).

### 2.3. Overexpression of ANAC046 Leads to an Increased Leaf, Silique and Seed Coat Permeability

**Figure 3:**
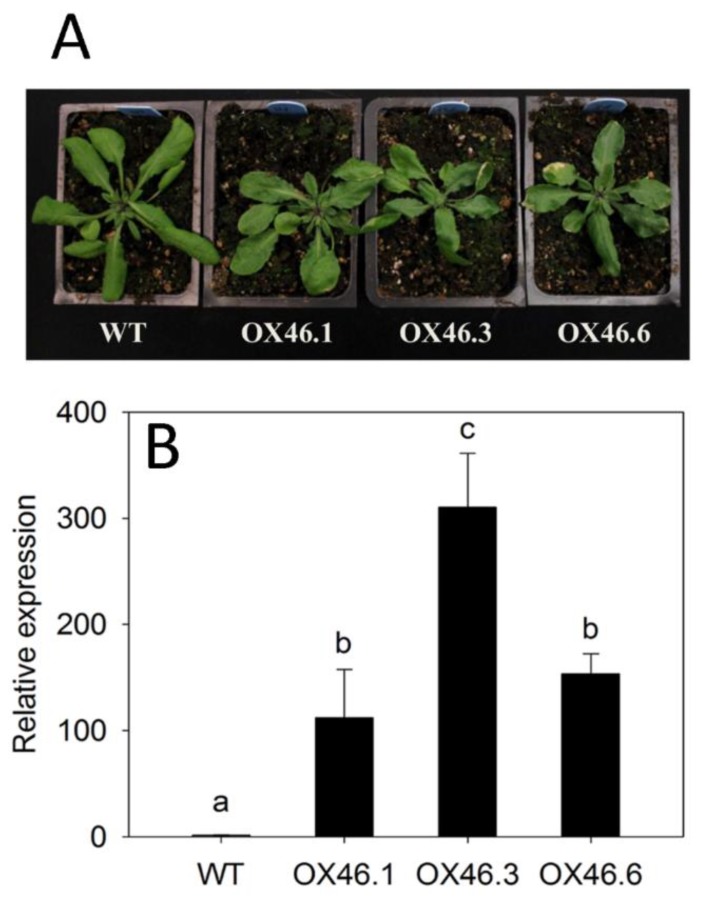
Generation of *ANAC046* overexpression (OX) transgenic plants. (**A**) Rosette leaves of the overexpression lines from 28-day-old plants show irregular growth pattern with patches of wounds or premature senescence on the edges of the leaves. (**B**) Quantitative RT-PCR analysis of *ANAC046* transcripts in the rosette leaves of three independent overexpression lines. *GAPDH* was used as an internal control to normalize the values of target transcripts. Relative expression values represent the means ± SD from three biological replicates (*n* = 3). Data were analyzed using one-way ANOVA LSD test (*p* < 0.05).

We employed gain-of-function approach to determine the physiological role of *ANAC046* in *Arabidopsis*. Eight independent overexpression lines (35S::*ANAC046*) were obtained in T1 generation. From these eight independent lines, three lines were found to have a single insertion of transgene copy as analyzed through segregation analysis of T2 generation. These putative overexpression lines (OX46) exhibited growth defects characterized by relatively smaller plant statures, slightly altered leaf shapes with rolling and occasional presence of wound patches on the surfaces than WT (Figure 3A). These effects were found to be correlated with the level of transgene expression in the independent overexpression lines. Quantitative RT-PCR of the three independent transgenic lines overexpressing *ANAC046*; OX46.1, OX46.3 and OX46.6, showed that these lines highly expressed *ANAC046* transgene to varying levels compared to the WT plants (Figure 3B).

**Figure 4:**
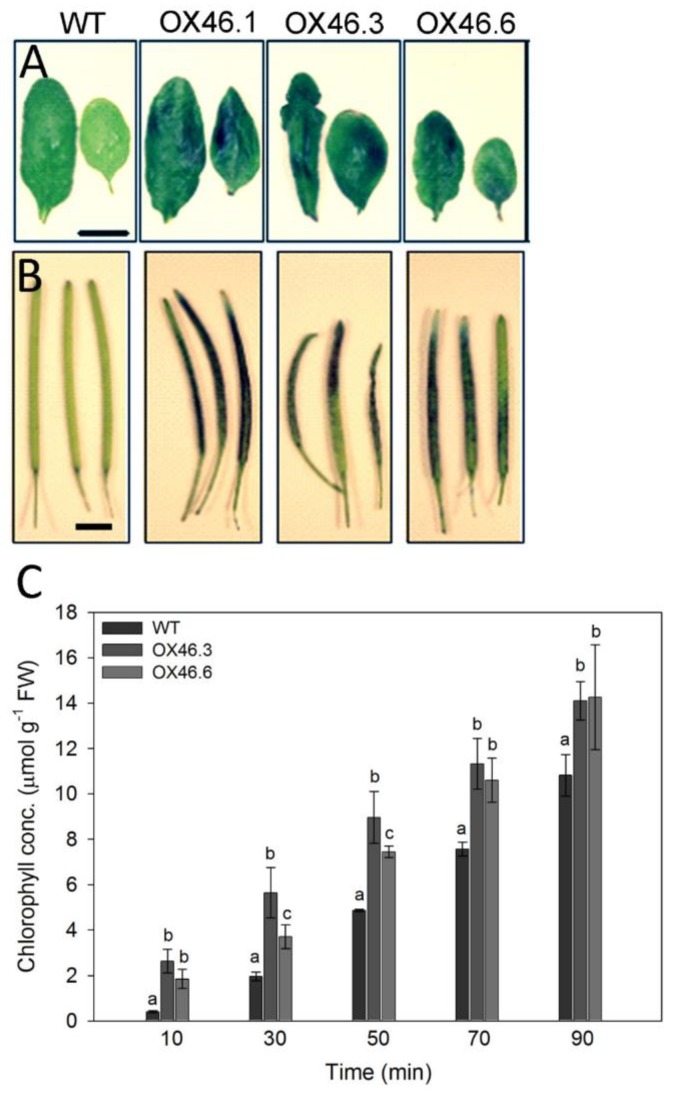
Surface/cuticle permeability of leaves and siliques tested with Toluidine Blue O (TBO). Absence/presence of blue staining of (**A**) rosettes leaves and (**B**) siliques of WT, *ANAC046* overexpression (Bars, A = 0.5 cm, B = 0.2 cm). Leaves from 21-day-old and siliques from 50-day-old were detached from the plant and incubated in 0.1% (*w*/*v*) TBO staining solution for two min, followed by washed with de-ionized water for three times. Leaves and siliques from *ANAC046* overexpression lines stained blue (Bar = 0.2 cm). (**C**) Chlorophyll leaching assay of rosette leaves. Whole rosettes from 35-day-old plants were harvested, weighed and subjected to analysis. Data represent the means ±SD of three biological replicates. Data were analyzed using one-way ANOVA LSD test at *p* < 0.05.

We, therefore, decided to investigate the basis of the pleiotropic growth defects in the overexpression lines. Once soaked in a toluidine blue O (TBO) staining solution, the leaves of overexpression lines clearly exhibited an increased permeability/penetration of the stain into the leaf through the cuticle as demonstrated by the localized blue-colored patches of the leaves (Figure 4A). The siliques of the *ANAC046* transgenic lines also demonstrated similar TBO staining patterns (Figure 4B). The altered leaf surface/cuticular permeability was further analyzed by performing chlorophyll leaching assay. Consistent with TBO permeability, *ANAC046* overexpression lines exhibited significantly increased chlorophyll leaching rates compared to the WT plants (Figure 4C). This suggests that ectopic expression of *ANAC046* leads to an overall defective surface phenotype in *Arabidopsis thaliana*.

### 2.4. ANAC046 Overexpression Leads to an Enhanced Expression of Suberin Biosynthesis Genes in Rosette Leaves

**Figure 5:**
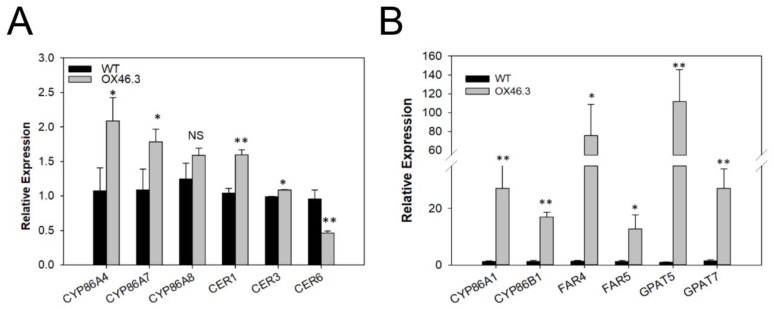
Quantitative RT-PCR analysis of cutin and suberin biosynthesis genes. qRT-PCR analysis of (**A**) cutin biosynthesis and (**B**) suberin biosynthesis genes in the rosettes leaves of 21-day-old WT and *ANAC046* overexpression line. *ACT7* was used as the internal control. Data represent the means ±SD values from two technical and three biological replicates and were analyzed statistically using Student’s t-test (*p* ** < 0.01, *p* * < 0.05, NS—not significant).

The increased permeability for dyes in different tissues of the *ANAC046* lines suggested a defective cuticle layer/structure [41]. This prompted us to investigate the cuticular properties of *ANAC046* transgenic lines, including the analysis of transcript levels of well-characterized genes involved in cuticle biosynthesis of *Arabidopsis*. The qRT-PCR analysis showed that the transcript levels of *CYP86A4*, *CYP86A7* and *CER1* were slightly higher in *ANAC046* overexpression line compared to WT and that of *CER6* were lower. The transcript levels of *CYP86A8* were similar to WT (Figure 5A). This suggested that the expressions of cutin biosynthesis genes were not largely affected by *ANAC046* overexpression. Recently, a gain-of-function study in *Arabidopsis* revealed that overexpression of *AtMYB41* resulted in the ectopic biosynthesis of suberin-like monomers in the leaf tissues [26]. These overexpression lines also had defective leaf-surfaces and revealed an increased permeability for stains and greater chlorophyll leaching. We, therefore, also analyzed the expression levels of the well-characterized genes involved in suberin biosynthesis in the rosette leaves of WT and *ANAC046* transgenic line. The qRT-PCR analysis revealed that the transcript levels of the well-characterized suberin genes, such as *CYP86A1*, *CYP86B1*, *FAR4*, *FAR5*, *GPAT5* and *GPAT7*, were drastically induced in the overexpression line compared with the WT (Figure 5B). This suggests that the *ANAC046* overexpression may have increased suberin biosynthesis in the rosette leaves in *Arabidopsis thaliana*.

### 2.5. Overexpression of ANAC046 Promotes Deposition of Leaf Wax in Arabidopsis 

**Figure 6:**
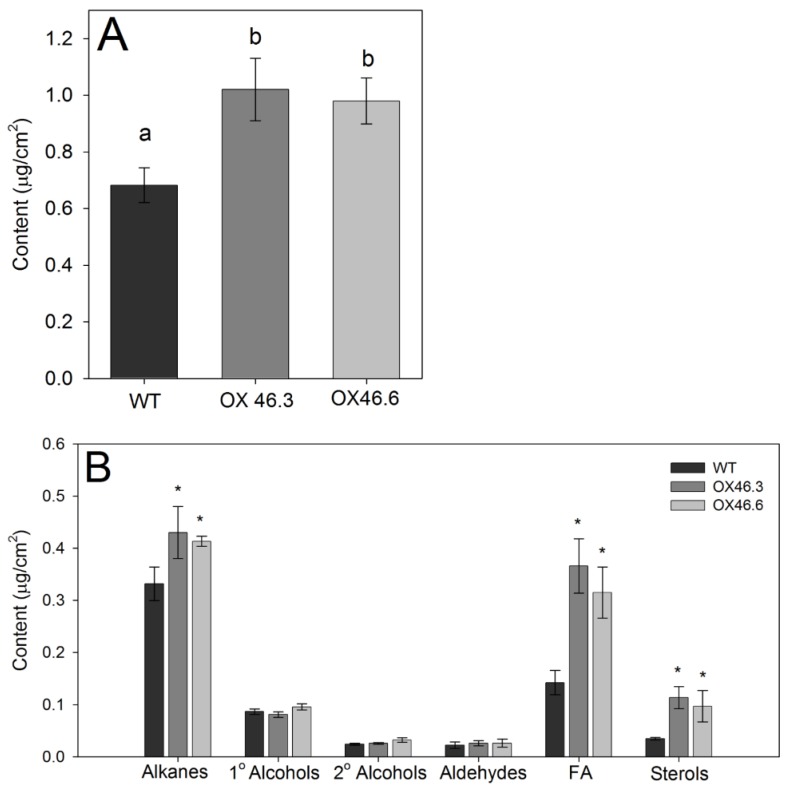
Wax analysis of *Arabidopsis* leaves. (**A**) Total wax and (**B**) wax constituents of the rosettes leaves of 21-day-old WT and *ANAC046* overexpression lines, extracted by immersing leaves in CHCl3 for 10 s and analyzed using gas chromatography and mass spectrometry. Absolute amounts and wax constituents/monomer composition are given as means in µg per cm^2^ ± SD for 4 replicates (*n* = 4 leaves). Data were analyzed using ANOVA, Tukey test. * indicate significant differences at *p* < 0.05 level.

Not only suberin, but also lipophilic leaf cuticular waxes make strong barriers for water and nutrient transport as well as block the pathogen entry into the plant. Hence, we analyzed the composition and content of cuticular waxes in the rosette leaves of WT and *ANAC046* overexpression lines. The overexpression lines (OX46.3 and OX46.6) had approximately 30–35% greater content of leaf wax than the WT (*p* < 0.05; Figure 6A). Regardless of the genotype, the leaf waxes of *Arabidopsis* primarily consisted of the substance classes of alkanes, primary (1°) and secondary (2°) alcohols, fatty acids (FA), aldehydes, and some sterols (Figure 6B). In all genotypes, alkanes and fatty acids were the prominent components in the leaf wax. However, the OX46.3 and OX46.6 lines had significantly greater content of FA (C_24_ and C_26_), sterols (β-Sitosterol) and alkanes (C_29_) than that of WT but it was more pronounced for fatty acids and sterols (*p* < 0.05; Figure 6B; Supplementary S2B). Chain lengths of these wax components ranged from C_16_ to C_34_ with strong predominance of longer carbon chain lengths, C_29_ or beyond (Supplementary Figure S2A). The most prominent chain lengths were the C_26_, C_29_, C_31_ and C_32_. In comparison, the overexpression lines of OX46.3 and OX46.6 had significantly greater content of C_24_, C_26_ and sterols than that of WT (red boxes), but it was the reverse for the chain lengths of C_31_–C_34_ (blue box) (Supplementary Figure S2A,B). 

### 2.6. Overexpression of ANAC046 Resulted in Increased Root Suberin Content

**Figure 7:**
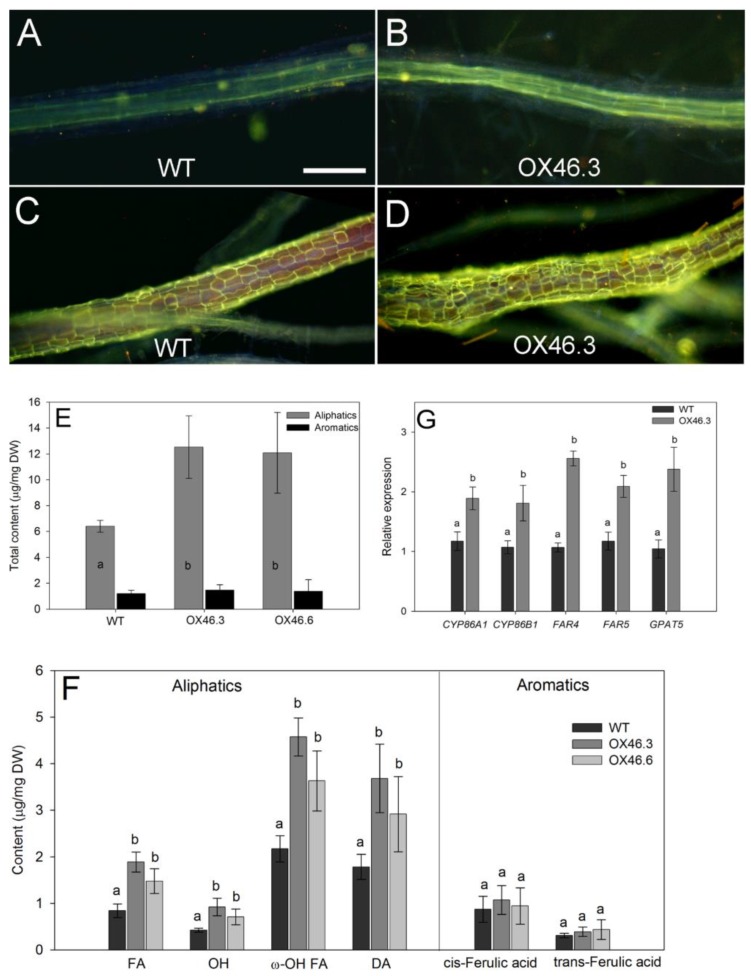
Suberin deposition in *Arabidopsis* roots. Histochemical detection of suberin accumulation in the endodermis (**A**,**B**) and periderm (**C,D**) of roots of 21-day-old WT and *ANAC046* overexpression line, stained with fluochrome, Fluorol Yellow O 88. Yellowish/greenish fluorescence indicates lipophilic suberin in the endodermis and periderm of roots. Bars = 100 µm (**A,B**) and 250 µm (**C,D**). (**E**) Total suberin contents and (**F**) substance classes of aliphatic and aromatic suberin in the roots of three-week-old WT and *ANAC046* transgenic lines. Enzymatically digested and solvent-extracted root cell walls were subjected to BF3/MeOH transesterification. Total suberin and substance classes were analysed using GC-FID and GC-MS. Absolute amounts and suberin monomers are given as means in µg per mg DW ± SD for 4 roots (*n* = 4). (**G**) Quantitative RT-PCR analysis of suberin biosynthesis genes in the roots of 21-day-old WT and *ANAC046* transgenic lines. *ACT7* was used as the internal control. Expression values are the means of two technical and three biological replicates. Data were analyzed using ANOVA, Tukey test. Different letters indicate significant differences at *p* < 0.05 level.

Since, *ANAC046* is predominantly expressed in the endodermis and periderm of roots—the main sites of suberin biosynthesis and deposition, we analyzed if *ANAC046* overexpression could alter suberin biosynthesis and deposition in *Arabidopsis* roots. The suberin lamellae, stained with lipophilic fluorochrome, Fluorol Yellow 088, were detected as bright yellow stains in the cell walls (Figure 7A–D). In both WT and OX46 transgenic lines, suberin lamellae deposition started around 10 mm from the root tip. In OX46.3 line, the endodermal suberin lamellae stained markedly brighter than the WT at 50% of the total length of respective roots (Figure 7A *versus* Figure 7B). The periderm of roots was indicated by a mesh of yellowish green fluorescence (Figure 7C,D) and appeared only at the very basal parts of the roots. The stained periderm was markedly brighter in OX46.3 line than that of WT (Figure 7C *versus* Figure 7D). Biochemical analysis of the suberin content in the roots of WT and OX46 overexpression lines showed the presence of both aliphatic and aromatic suberin where the content of aliphatic suberin was markedly greater than the released aromatic suberin (Figure 7E). Interestingly, in comparison, the total content of aliphatic suberin in the overexpression lines was approximately twice the content of WT. However, the total content of aromatic suberin was same in both WT and overexpression lines (Figure 7E).

The detailed study of the monomer composition of aliphatic suberin in the roots of WT and OX46 transgenic lines revealed that it was mainly composed of fatty acids (FA), ω-hydroxy acids (ω-OH), α,ω-dicarboxylic acids (DA), and alcohols (OH), and amongst these components, ω-hydroxy acids and DAs were the most prominent (Figure 7F). Interestingly OX46.3 and OX46.6 had significantly greater amounts of all components compared to WT (*p* < 0.05; Figure 7F). However, the cis- and trans-ferulic acids content which constitute the aromatic suberin, were the same in WT and overexpression lines. Further analysis of the individual aliphatic monomer components of suberin revealed that C_22_ fatty acids, C_18(1)_ and C_22_ diacids, and C_18(1)_ ω-hydroxy acids were the prominent components in both WT and OX46 transgenic lines (Figure S3). However, the overexpression lines had significantly greater amounts of these monomers as well as other monomers, such as C_16_, diacids and all monomers of ω-hydroxy fatty acids, than that of WT (*p* < 0.05; Supplementary Figure S3).

To correlate with the suberin biochemical studies, the expressions of the well-characterized suberin biosynthesis genes, such as *CYP86A1*, *CYP86B1*, *FAR4*, *FAR5* and *GPAT5* were determined in the root tissues of WT and *ANAC046* overexpression line. qRT-PCR analyses confirmed that all these genes were significantly induced in the roots of overexpression line compared with the WT (Figure 7G). 

## 3. Discussion

Although significant progress has been made in elucidating the suberin biosynthetic pathway in last few years, especially in the model species *Arabidopsis*, the knowledge about the transcriptional regulation of this pathway is still very limited. In this study, we have identified a NAC transcription factor in *Arabidopsis*, *ANAC046*, which promotes the accumulation of suberin in roots, specifically in the endodermis of primary roots and the periderm of secondary roots. This finding is a new addition to the previously identified *StNAC103*, a member of NAC TFs family, which has been identified as a negative regulator of suberin accumulation and associated waxes in tuber skins [30]. In addition to these NAC transcription factors, recent studies have identified a few members of MYB TFs family which positively regulated suberin biosynthesis in *Arabidopsis* [26,27,28], and cork oak tree [29], in spatio-temporal manners.

The function of *ANAC046* in *Arabidopsis* was analyzed by using gain-of-function approach, in which the coding region of *ANAC046* was overexpressed in *Arabidopsis* under cauliflower mosaic virus 35S promoter (35S:*ANAC046*). The resulting *ANAC046* overexpression lines showed defective leaf surfaces as demonstrated by increased permeability of their rosettes leaves for TBO stain and increased chlorophyll leaching rates (Figure 4A,C). The defective-surface phenotype exhibited in overexpression lines was not restricted to rosette leaves only. A similar increase in the permeability for TBO stain was found in the siliques of *ANAC046* overexpression lines, (Figure 4B). Since mutants defective in cuticle layer/cutin biosynthesis often exhibit defective leaf-surface phenotypes [42], we hypothesized that cutin biosynthesis was altered in *ANAC046* overexpression lines. However, a detailed qRT-PCR analysis of cutin biosynthesis genes revealed that they were not altered at large, rather the genes related to suberin biosynthesis were upregulated/activated in the rosettes leaves of 

*ANAC046* overexpression line (Figure 5A vs. Figure 5B). These included (1) *CYP86A1*—involved in the synthesis of omega-hydroxyacids with a chain length of <C20 in root endodermis [18], (2) *CYP86B1* —involved in the synthesis of C22- and C24-hydroxyacids and alpha, omega-dicarboxylic acids, in roots and seeds [19], (3) *FAR4* and *FAR5* - involved in the synthesis of primary fatty alcohols, C20:0-OH and C18:0-OH, respectively, in root, seed coat, and wound-induced leaf tissue [16], and (4) *GPAT5* and *GPAT7*—involved in the metabolism of suberin-related glycerolipids [21].

Cuticular waxes are part of the leaf cuticle composed of a mixture of linear, long-chain aliphatic molecules with different chain lengths [43] and known to establish the barrier for cuticular transport [12,44]. In this study, the detailed biochemical analysis of the cuticular wax revealed that an overall increase in the leaf wax content in overexpression lines compared to WT (Figure 6A). Detailed monomer analysis revealed that typical wax monomers, such as alkanes and fatty acids together with sterols were responsible for this increment (Figure 6B). This increase in alkanes is supported by the observation that expressions of *CER1* and *CER3*, which are involved in the biosynthesis of long-chain alkanes, were elevated in overexpression line compared with WT (Figure 5A). This is different from the findings of Kosma et al. (2014) in which overexpression of *AtMYB41* led to the accumulation of suberin-associated leaf waxes, such as alkyl hydroxycinnamates, monoacylglycerols (MAG) and phenylpropanoids (PP) in *Arabidopsis*. The carbon chain length distribution analysis of the cuticular waxes further showed a significant increase of long carbon chains (C24, C26) of overexpression lines compared to WT (Supplementary Figure S2). Interestingly, there was a reduction in the very long carbon chains (C31-C34) of overexpressed lines, which correlated with reduced expression of *CER6* in the overexpression line, which is involved in the synthesis of VLCFA (C > 30) (Supplementary Figure S2). 

Given the increase in lipophilic (hydrophobic) cuticular waxes in overexpression lines compared with WT, it was very intriguing to find that leaves of overexpression lines were more permeable to water-based stain of TBO than WT (Figure 4A). This was also true for siliques. Greater leaf permeability of overexpression lines was further supported by significantly higher chlorophyll leaching from the leaves (Figure 4C). The increased permeability to TBO stain and chlorophyll leaching may have resulted either due to cuticular cracks or due to localized cell death on the leaf surfaces. In both cases, the sealing property of the cuticle layer should be comprised. The later possibility is of particular relevance in the light of two recent studies in which *ANAC046* was shown to promote senescence in leaves [39] and programmed cell death in root cap [40]. Both studies identified several genes (such as *BFN1*, *EXI1*, and *RNS3*) which are known to promote programmed cell death in *Arabidopsis*. The inducible expression of *ANAC046* completely arrested the growth of young *Arabidopsis* seedlings which eventually culminated in their death [40]. In our study, the constitutive overexpression lines also showed the visible signs of premature senescence and or necrosis of the leaves and siliques during the later growth phase, both in intact plants and in dark-treated detached leaves (Supplementary Figure S1B–D), as reported by Oda-Yamamizo et al. [39]. In this context, it is likely that *ANAC046* is promoting suberin deposition concurrently with programmed cell death to seal the damaged area to ensure tissue integrity and to further check the progression of cell death to neighboring cells. An analogous phenomenon has been observed in lace plant (*Aponogeton madagascariensis*), in which the formation of perforations during leaf expansion is facilitated by programmed cell death of distinct patches of cells which then coincides with suberin deposition, and other cell wall modifications [45,46]. However, further studies will be required to confirm this in *Arabidopsis thaliana*.

The plant secondary biopolymer, suberin is primarily deposited in the endo- and exodermis of plant roots, and periderms including wound periderm [2,3,9,20]. Suberized apoplastic barriers are known to (1) decrease the movement of water and solutes into the roots/vascular tissue, (2) restrict pathogens entry and (3) heal/seal the wounded areas in the plant tissues [6,7,11]. Although the components of the enzymatic pathway involved in the biosynthesis of suberin polymers have been identified in the past decade [1], the transcriptional network regulating the spatio-temporal of suberin is still largely unknown. To date, only a few members belonging to MYB family of transcription factors have been identified to regulate the biosynthesis and deposition of suberin polymers in *Arabidopsis thaliana*. For example, two *Arabidopsis* homologous genes, *MYB107* and *MYB9*, have been found to positively regulate suberin biosynthesis in seed coats of *Arabidopsis* [27,28]. These genes are known to coordinate the production of aromatic monomers through the phenylpropanoid pathway, while activating aliphatic monomer production in fatty acid biosynthesis as well as the extracellular transport and polymerization of these aromatic and aliphatic monomers. The members of the neighboring clade of MYB transcription factors that include *AtMYB16* and *AtMYB106* have been demonstrated previously to regulate cutin biosynthesis [47], whereas, *AtMYB74* and *AtMYB102* have been found to be involved in the plant’s response to wounding, salt, and osmotic stresses [48,49]. In another study, overexpression of *MYB41* in *Arabidopsis* resulted in ectopic deposition of suberin in the rosette leaves [26]. Since its expression was only detectable during stressful growth conditions, *AtMYB41* was proposed to have a role in the promotion of suberin biosynthesis during stresses (stress-induced suberin). In the present study, we have found the role of *ANAC046* in *Arabidopsis thaliana* in the promotion of suberin biosynthesis in roots. Moreover, *ANAC046* expression was induced upon mechanical wounding as revealed by increased gus activity in the rosette leaves of P_ANAC046_:GUS lines, and it is possible that *ANAC046* may also be involved in wound-induced suberin biosynthesis in leaves.

In plants, biosynthesis and deposition of suberin biopolymers is stringently regulated in a spatio-temporal manner [3,4]. Suberin polymer is composed of aliphatic and aromatic domains, and mainly synthesized and deposited in the roots [14,16,20,21]. The expression analysis of the P_ANAC046_:GUS lines revealed that *ANAC046* is mainly expressed in the roots, more likely in the root endodermis which is the primary site of suberin deposition in *Arabidopsis*, which lacks an exodermis [9]. The endodermal suberin lamellae in the overexpression lines did not deposit earlier compared to the WT, but it is likely more suberin deposited into the lamellae of the overexpression lines which resulted in brighter stains (Figure 7B). This prompted us to analyze suberin in the roots of *ANAC046* transgenic lines. The quantitative biochemical analyses of suberin using sensitive GC-MS technique revealed that *ANAC046* overexpression lines deposited approximately twice the amount of WT (Figure 7E). The qualitative histochemical staining further supported this finding in which both the endodermis and root periderm had brighter staining in overexpression lines than that of WT (Figure 7A–D). The principal aliphatic suberin components, such as ω-hydroxyacids, α,ω-diacids and fatty acids [1,13] were all increased in overexpression lines (Figure 7F). In contrast, aromatic monomers such as coumaric and ferulic acids, which are the main components of the polyaromatic domain which supposedly cross-linked with polyaliphatic domain [12,13] were the same in WT and *ANAC046* overexpression lines. In contrast to our findings with *ANAC046*, the overexpression of *AtMYB41* did not alter the aliphatic suberin content or composition of roots or seed coats as it did in the leaves of *Arabidopsis* lines [26]. It clearly demonstrates that the transcriptional regulation of *ANAC046* is different from *AtMYB41* in suberin biosynthesis and deposition.

In this study, we have demonstrated the involvement of *ANAC046* in the promotion of suberin biosynthesis and deposition in *Arabidopsis thaliana*. Using a number of molecular, biochemical and anatomical/staining approaches, it is demonstrated that *ANAC046* enhanced the biosynthesis and deposition of aliphatic suberin in roots and leaves. This conclusion is supported by the observations of (1) pleiotropic surface defects and activation of suberin biosynthesis genes in young leaves, and (2) increased suberin deposition in root endodermis and periderm of *ANAC046* overexpression lines.

## 4. Materials and Methods 

### 4.1. Transcriptional Activity Assay of ANAC046 Using Yeast

The *ANAC046* coding sequences corresponding to the full-length protein (1–338aa), the N-terminal NAM domain (1–172aa), and the C-terminal domain (173–338aa) were amplified using primer pairs yNAC46-F and yNAC46-R; yNAC46-F and yNAC46N-R, and yNAC46C-F and yNAC46-R, respectively. The amplified fragments were then cloned into the pGBKT7-DB vector between NdeI and BamHI restriction sites. The constructs with correct sequences were transformed into Y2HGold strain using the LiAc yeast transformation method as described in Yeastmaker™ Yeast Transformation System 2 User Manual (Takara Bio, Mountain View, USA). Transcriptional activities of the corresponding constructs were determined by growing the yeast on selection plates, SD/Trp/X/A and SD/-His.

### 4.2. Subcellular Localization of ANAC046 

Subcellular localization of *ANAC046* was determined as described earlier by Mahmood et al., [50]. Briefly, the *ANAC046* coding sequence was first cloned into pENTR/D-TOPO vector and then transferred into CaMV 35S promoter-Gateway cassette plasmid to generate a transcriptional fusion at its 3′ end with GFP coding region using a LR recombination reaction (Invitrogen, Carlsbad, CA, USA). 35S:GPF (control) and 35S:*ANAC046*-GFP cassettes were transformed into tobacco Bright Yellow-2 (BY2) cells through biolistic method as described by Banjoko and Trelease [51]. Bombarded cells were fixed in formaldehyde after 6 h of incubation and then stained with DAP1 for 15 min. Localization of proteins was determined by visualizing the transformed cells using an epifluorescence microscope (Carl Zeiss Meditech AG, Oberkochen, Germany). 

### 4.3. Generation of ANAC046 Transgenic Plants

*ANAC046* overexpressor (35S:*ANAC046*) construct was generated by using Gateway technology (Thermo Fisher Scientific, Waltham, MA, USA). For this purpose, the PCR products were first amplified and cloned into the pDONR*™* 221 vector by using primer pairs, DNAC46-F and DNAC46-R, DNAC46-F, using a BP Clonase™ enzyme mix (Invitrogen, Carlsbad, CA, USA). Positive clones with correct sequences were used subsequently to shuttle the coding regions into the destination vector, pB7WG2D, by using LR Clonase™ enzyme mix. To generate P_ANAC046_:GUS transgenic lines, ~2.04 kb region upstream of the start codon (ATG) was amplified using the PNAC46-F and PNAC46-R primer pair and cloned into pDONR221 using a BP Clonase™ enzyme mix (Invitrogen^®^). The promoter region was then shuttled into the destination vector, pBGWFS7, to generate P_ANAC046_:GUS construct using LR Clonase™ enzyme mix (Invitrogen, Carlsbad, CA, USA). These destination vectors were then transformed into *Arabidopsis thaliana* (Col-0) through floral dip method by using *Agrobacterium*-mediated transformation [52]. The selection of homozygous transgenic lines through T_1_, T_2_ and T_3_ generations was performed as described by Mahmood et al. [50]. To determine the transcript levels of transgene in *ANAC046* transgenic lines, total RNA was isolated from the rosette leaves of 28-day-old soil-grown WT and *ANAC046* transgenic plants using Triazole (Triagent, Sigma-Aldrich, St. Loius, MO, USA) method. Primer pairs, qNAC046-F and qNAC46-R, were used in quantitative RT-PCR. *GAPDH* was used as an internal control to normalize the expression values of transgene transcript levels [53]. 

### 4.4. GUS Staining

GUS staining of the plant tissues, harvested from P_ANAC046_:GUS transgenic lines, was performed as described by Mahmood et al. [50]. Briefly, plant tissues at various developmental/growth stages were collected in chilled 90% acetone. These included roots from 12-day-old seedlings grown on 1/2 MS-agar (1% *w*/*v*) (Sigma-Aldrich, St. Loius, MO, USA) containing 1% sucrose; whole rosette, roots and inflorescence including floral buds and young siliques from 32-day-old plants; green and senescing siliques from 42-day-old plants; and rosette leaves from 21-day-old plants after 3, 6, 18 and 24 h of mechanical wounding. The collected samples were then vacuum infiltrated with GUS staining solution: 100 mM Sodium phosphate buffer (pH 7.2), 10 mM EDTA, 0.5 mM Potassium ferricyanide, 0.5 mM Potassium ferrocyanide, 2 mM X-Glu (5-bromo-4-chloro-3-indolyl-beta-D-glucuronic acid), 0.1% tritonX, 20% methanol. Plant tissues were incubated in the GUS staining solution at 37 °C overnight in darkness. GUS staining solution was then removed from the samples and tissues were treated with 70% ethanol to clear the chlorophyll and then photographed.

### 4.5. Toluidine Blue O Staining

The permeability of leaves for Toluidine Blue O (TBO) was performed as previously described by Tanaka et al. [54]. Leaves with cuticular cracks/wounds should exhibit intense blue stains/color due to rapid penetration of stain into the leaf, whereas, the cuticle of healthy, unwounded leaves should resist the dye movement into the leaf. Plant tissues, rosette leaves from 21-day-old and siliques from 50-day-old plants, were detached from WT and *ANAC046* transgenic lines and immersed in an aqueous solution of 0.05% (*w*/*v*) TBO for two mins. The TBO solution was then removed from the samples and plant tissues were washed gently with distilled water to remove excess TBO. Three to four whole rosettes were immersed in the staining solution representing each genotype.

### 4.6. Chlorophyll Leaching Assay

All genotypes had the same green phenotypes and there were no apparent differences in the greenness of leaves between the genotypes (Supplementary Figure S1A). Chlorophyll leaching assay was performed as described by Xia et al. [55]. Briefly, the entire rosette leaves representing each genotype were separated from the 35-day-old plants, weighed and incubated in 80% ethanol in dark with gentle shaking. Samples were obtained at several time points for each genotype and subjected to spectrophotometric analysis by recording absorbance of the samples at 664 and 647 nm (*n* = 3). Chlorophyll concentration in each sample was determined by using the following equation:

Total chlorophyll concentration (µmoles/mg fresh weight of sample) = 7.93 (A_664_) + 19.3 (A_647_).

### 4.7. Quantitative RT-PCR Analysis of Genes Related Suberin and Cutin Biosynthesis Pathway

To analyze relative expression of genes involved in cutin and suberin biosynthesis, total RNA was isolated from the rosette leaves of 21-day-old soil-grown WT and *ANAC046* transgenic plants using Triazole (Triagent, Sigma-Aldrich, St. Loius, MO, USA) method. For analysis in root tissues, WT and *ANAC046* transgenic lines were grown hydroponically for 21 days and root tissues were harvested for RNA extraction. RNA samples were treated with *RQ1 DNase* (Promega, Madison, WI, USA) to digest any residual genomic DNA and were subsequently cleaned with the RNeasy Kit (Qiagen, Hilden, Germany). RNA samples were then subjected to cDNA synthesis using first-strand cDNA synthesis kit (Quantabio, Beverly, MA, USA). Reaction mixtures were prepared containing equal amount of cDNA (50–100 ng) of each sample and SYBR^®^ Green dye (Quanta) according to the manufacturer’s instructions and analysed using the Applied Biosystems 7500/7500 Fast Real-Time PCR System (Waltham, MA, USA). Actin 7 (*ACT7*) was used an internal control to normalize the expression values of target genes. Primers sequences for the analyzed genes are provided in supplementary Table S1.

### 4.8. Analysis of Wax Composition in Leaves 

Wax composition and content from freeze dried leaves were analyzed by gas chromatography and mass spectrometry. Before freeze drying and extracting wax, the leaves were scanned to measure their surface areas. For total wax extraction, *Arabidopsis* leaves (fully-expanded 3rd to 5th leaves from the top) were excised from 21-day-old WT and *ANAC046* transgenic plants, and immersed in 2 mL CHCl_3_ at room temperature for 10 s. Every wax sample was directly spiked with 50 µL *n*-tetracosane (C24), serving as an internal standard. Chloroform was evaporated under a gentle stream of N_2_ at 60° C to a final volume of 200 μL. Samples were then derivatized using 20 µL BSTFA (*N,O*-bis(trimethylsilyl)-trifluoroacetamid) and 20 µL Pyridine. Quantification of wax molecules using the GC-FID (GC-FID; CG-Hewlett Packard 5890 series H, column: 30 m DB-1 i.d. 0.32 mm, film 0.2 µm; J&W Scientific) system was performed by on column injection of 1 µL of each sample. Identification of the single wax molecules by GC-MS (GC-MS; quadrupole mass selective detector HP 5971, Hewlett-Packard) was done by comparing the obtained mass fragments with fragments listed in a homemade data library. 

### 4.9. Suberin Detection/Staining in the Roots Using Fluorol Yellow 088

In order to detect the suberin lamellae in the endodermis, periderm and other ectopic suberin in other cell walls (if present), whole roots from 21-day-old plants were cleared with 1% (*w*/*v*) NaOH overnight as previously described by Peterson and co-workers [56,57]. This method successfully removed cell wall polysaccharides and cleared the roots. The whole roots were then stained with lipophilic fluorochrome, fluorol yellow 088, for 1 h and viewed under an epifluorescence microscope using an ultraviolet filter set (excitation filter BP 365, dichroic mirror FT 395, barrier filter LP 397; Zeiss, Oberkochen, Germany). The aliphatic component of suberin in root endodermis (younger root zones with a primary growth) and periderms (mature, basal zones with a secondary growth) can be detected by yellow fluorescence under ultraviolet light as described by Brundrett et al. [58]. For comparison between genotypes, photographs were taken at 40 mm from the root tip to detect the suberin lamellae in the endodermis, and very base of the root to detect peridermal suberin. Since the root lengths of all genotypes were the same, the staining results were comparable. Three to four whole root systems from each genotype were used for suberin staining. 

### 4.10. Analysis of Suberin in Roots of Arabidopsis

To analyze suberin, the roots were harvested from 21-day-old plants and were incubated in an enzymatic solution as previously described by Zeier and Schreiber [59]. The digested root samples were washed in borate buffer (0.01 M; pH 9.2) for three days, replacing the solution every day, followed by three washes with deionized water. Then, the samples were thoroughly extracted in a mixture of chloroform and methanol (1:1; *v*/*v*) for three days, replacing the solution several times. After that roots were dried on Teflon discs and the individual weight was estimated. The resulting cell wall material was used for suberin analysis. Transesterification and identification of suberin monomers were carried out as previously described by Zeier and Schreiber [59]. Transesterification of purified wall materials, releasing suberin monomers, was carried out according to Kolattukudy and Agrawal [60]. GC analysis and MS identification of the derivatised degradation products were performed as previously described in detail by Zeier and Schreiber [59]. The results of the suberin analyses were related to the unit dry weight of the roots. Four replicates were used for each genotype. 

## Supplementary Materials

Supplementary materials can be found at https://www.mdpi.com/1422-0067/20/24/6117/s1.

## Abbreviations

GUS	β-glucuronidase
NAC	*NAM* (no apical meristem), *ATAF1,2* and *CUC2* (cup-shaped-cotyledon)
SAGs	Senescence-Associated Genes
