# Supplementary material for: Overexpression of ANAC046 Promotes Suberin Biosynthesis in Roots of Arabidopsis thaliana
Source: Int J Mol Sci. 2019 Dec 4;20(24):6117. doi: 10.3390/ijms20246117 (PMC6940730; doi:10.3390/ijms20246117)
Supplement: Supplementary file 1 [file ijms-20-06117-s001.pdf]

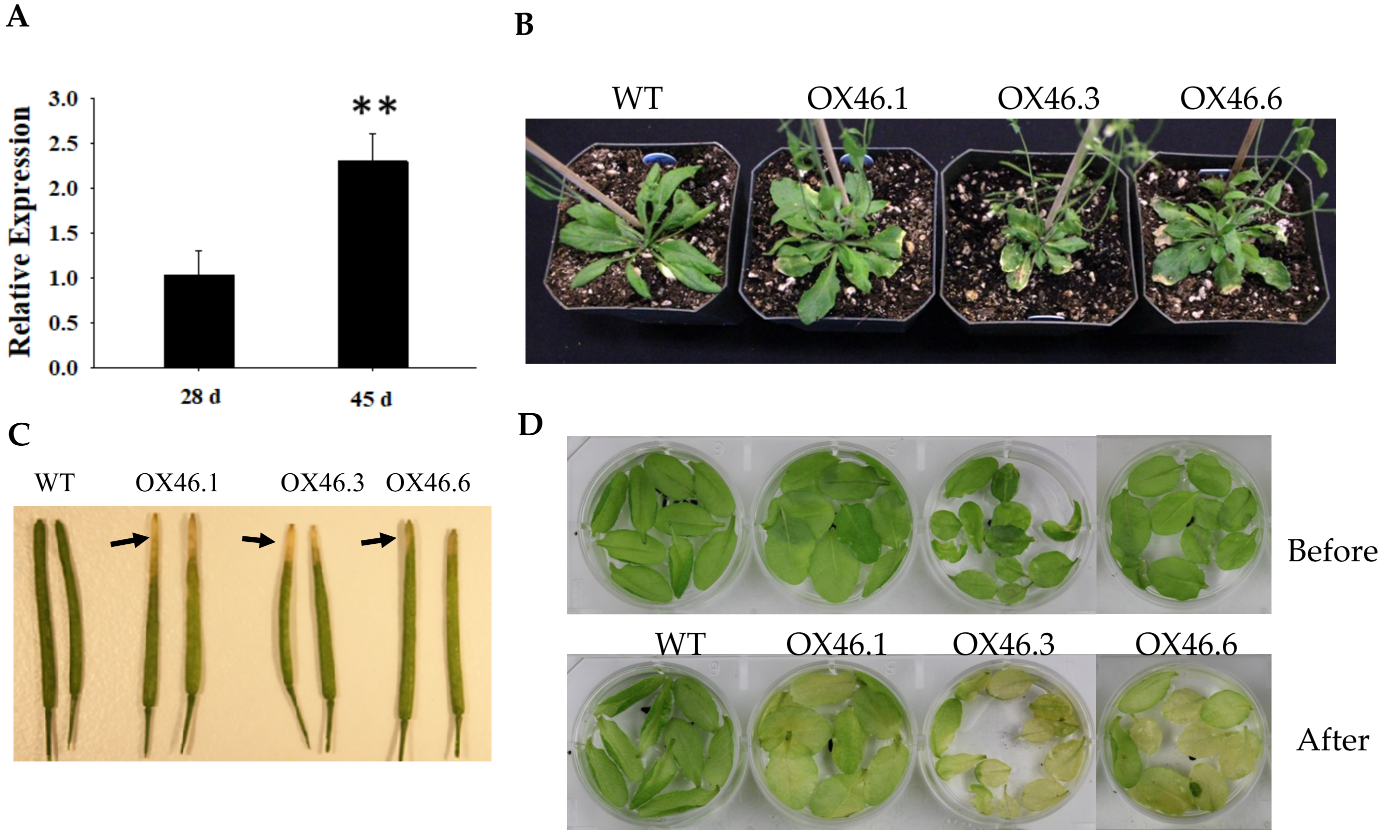

Figure S1: Senescence phenotype of ANAC046 Overexpression (OX46) lines. **(A)** Quantitative RT-PCR analysis of ANAC046 in the fifth leaf of Arabidopsis at 28 and 45 DAS. Values are the means (±SD) of two technical and three biological replicates. Data were analysed using Student's t-test (P**< 0.01). *ACT7* was used as an internal control. **(B)** Phenotype of WT and ANAC046 overexpression at 42 DAS (days after sowing). Overexpression lines clearly exhibit patches of wounds and premature senescence on their rosette leaves. Siliques of overexpression lines clearly exhibit symptoms of premature senescence on their apexes compared with WT. **(D)** Dark-induced leaf senescence in detached leaves of WT and ANAC046 overexpression lines. Three-week-old detached leaves were suspended in deionized water and incubated in dark for three-and-a-half days and then photographed.

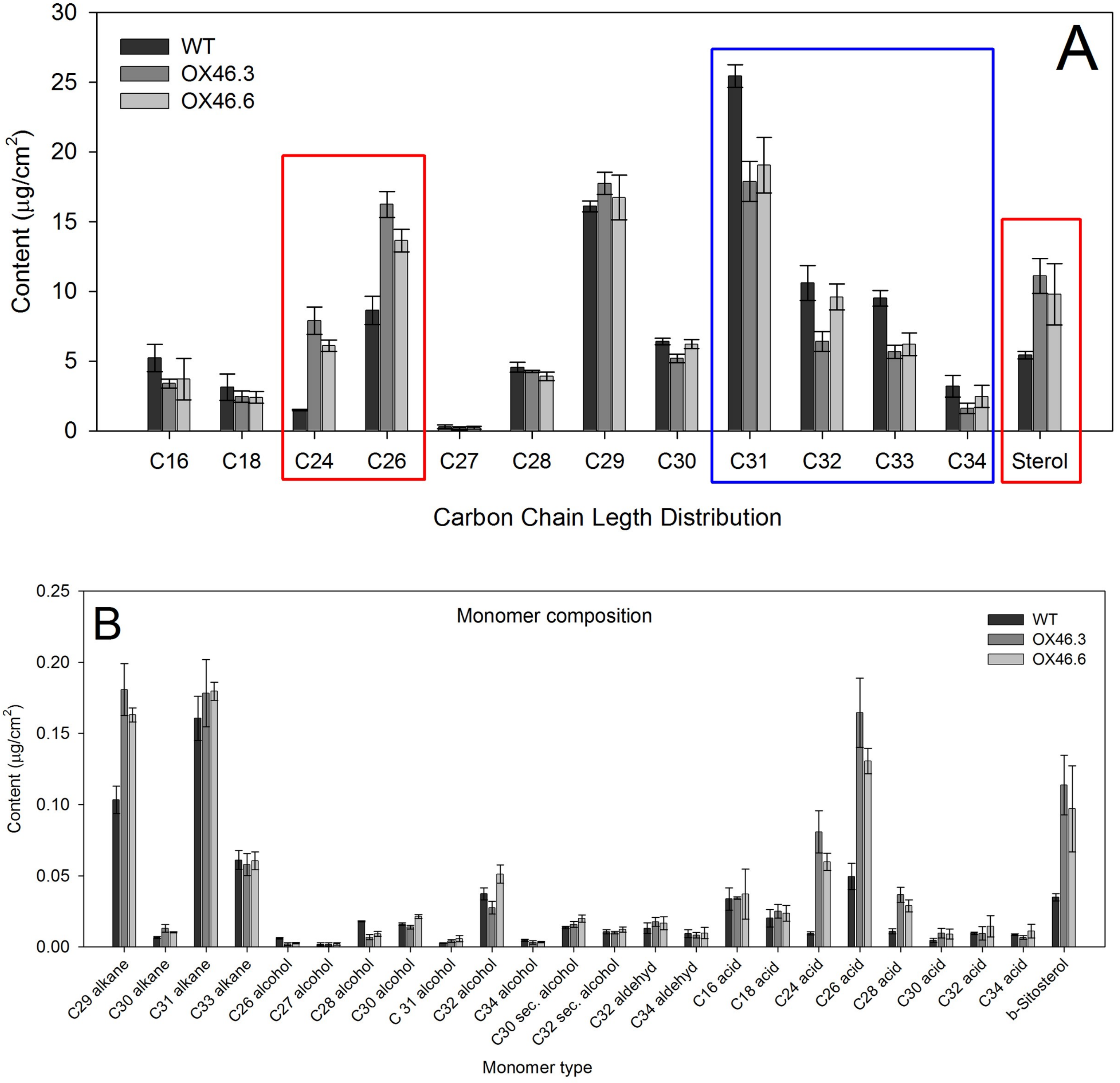

Figure S2: A) Carbon chain length distribution of leaf waxes. **B)** Monomer composition of leaf waxes. Total wax of the rosettes leaves of 21-day-old WT and ANAC046 transgenic lines were extracted by immersing leaves in $CHCl_3$ at 60° C for 20 s and analysed using GC and MS. The chain length distribution of waxes is given as means in µg per cm^2 ±SD for four leaves (n=4 leaves).

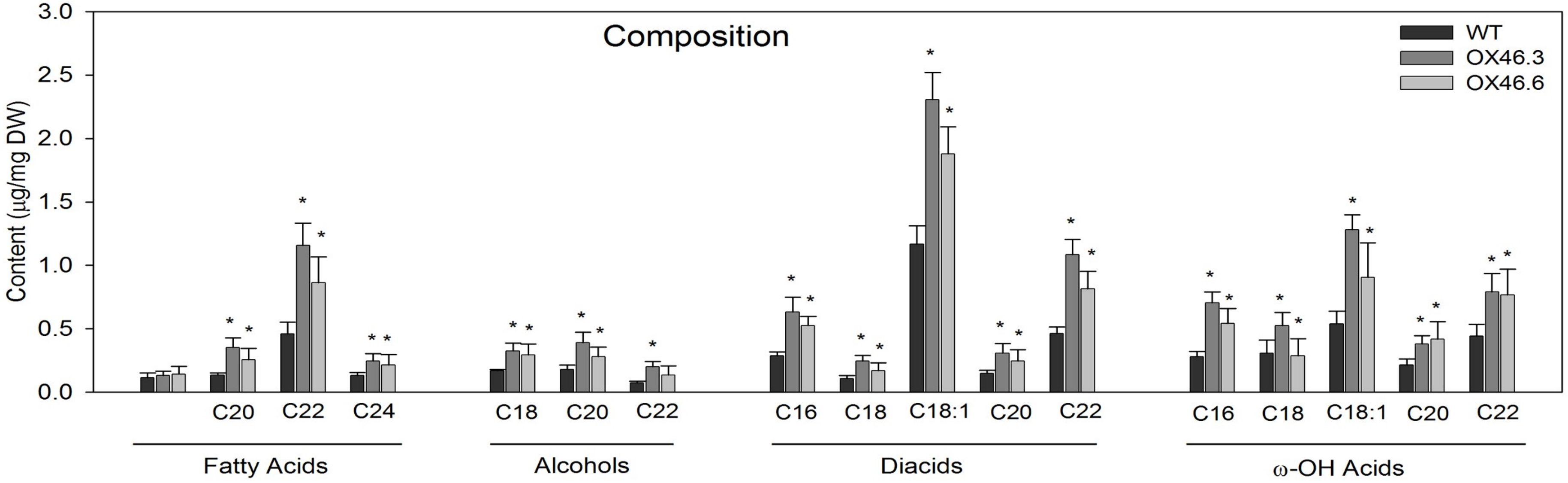

Figure S3: Monomer compositions of aliphatic suberin in the roots of 4-week-old Arabidopsis WT and ANAC046 transgenic plants. Enzymatically digested and solvent-extracted root cell walls were subjected to BF3/MeOH transesterification. Aliphatic monomers were analysed using gas chromatography and mass spectrometry. Absolute amounts of suberin monomers are given as means in µg per cm^2 ±SD for four roots (n=4). The statistical analysis was done for each group separately and compared with WT. The stars indicate significant differences at $P < 0.05$ level (one-way ANOVA, Tukey test).

Table S1. List of Primers used in the study

Oligo Name	Oligo Sequence
	5' → 3'
	**Primers used in transcription activity assay**
**yNAC46-F**	AGAATTCCATATGATGGTGGAAGAAGGCG
**yNAC46-R**	CGGGATCCTTAGCTAGTATATAAATCTTCCCAGAAGATC
**yNAC46N-R**	CGGGATCCTCAGTTCTTGTGAAAAACCCTAC
**yNAC46C-F**	AGAATTCCATATGGCTCCTAGTACTACAATCACTACTAC
	**Primers used to generate transgenic lines (35S:ANAC046 and PANAC046:GUS)**
**DNAC46-F**	GGGGACAAGTTTGTACAAAAAAGCAGGCTTTATGGTGGAAGAAGGCGG
**DNAC46-R**	GGGACCACTTTGTACAAGAAAGCTGGGTTTTAGCTAGTATATAAATCTTCCCAGAAGATC
**PNAC46-F**	GGGGACAAGTTTGTACAAAAAAGCAGGCTTTCCCAACTAGTAGTCATCATATCC
**PNAC46-R**	GGGACCACTTTGTACAAGAAAGCTGGGTTCTATATATATGTATGCTTGATCAAGA
**AttB1**	GGGGACAAGTTTGTACAAAAAAGCAGGCTTT
**AttB2**	GGGACCACTTTGTACAAGAAAGCTGGGTTT
	**Primers used in quantitative RT-PCR analysis**
**qNAC46-F**	AACCGCAACGCCAGAGATATC
**qNAC46R**	ACACGCTGACTTGCTACCATCC
	**Cuticle Biosynthesis genes**
**CYP86A4 -F**	TCGACCGTTTGGTTTACCTG
**CYP86A4 -R**	CCGATGGGTAAAGCCTGAG

**CYP86A7 -F**	CCACGTGTCTGGCCCTTA
**CYP86A7 -R**	CATGCGGTGAGCATTTGTTA
**CYP86A8-F**	ACCAGCCTAGAGCAGGAACA
**CYP86A8-R**	TAGGGTATGTGAAGCATGAGATG
**CER1-F**	CACCTCTTTCCTCCCCTCA
**CER1-R**	GTGGTGCAGCGAGTGGTAT
**CER3-F**	TTCTTGACTGGAGCCACTTCT
**CER3-R**	ACGGCGACAAAGGTAAAGAG
**CER6-F**	GACGTCGACATCCTTATCGTC
**CER6-R**	TAGCTGAGAGCGATGGTGTG
	**Suberin Biosynthesis genes**
**CYP86A1-F**	TTCTTCGGTGGCCTTGAG
**CYP86A1-R**	GTTTCCACCTCCCGGTTATT
**CYP86B1-F**	ATTCAACGCGGATGATGAA
**CYP86B1-R**	TGAAACTCAATGCTCGCTGT
**FAR4-F**	GGTTTCCTCGCCAAAGTGT
**FAR4-R**	TGCATGGCTGATTCATTGTC
**FAR5-F**	CATGAAAGAACTCGGAATGGA
**FAR5-R**	CAAGAAGCATTTCTCCCATTG
**GPAT5-F**	AGCTTCCTATGGAGGCAACA
**GPAT5-R**	GAACATAGTTCGCCACGTCA
**GPAT7-F**	GGATTCTCCAACTTTCCTATCTTTATG
**GPAT7-F**	TGGTTATGACCGTTGTAGTTCG
	**Internal Control**
**ACT7-F**	TGCACCGCCAGAGAGAAAAT
**ACT7-R**	TGAGGGATGCAAGGATTGATC
**GAPDH-F**	CTTGGAAGGAGCTAGGAATTGACA

**GAPDH-R**	ATGTGTTTCCCTGCACCTTCTC
